# Effect of *n*-3 Polyunsaturated Fatty Acid Supplementation on Metabolic and Inflammatory Biomarkers in Type 2 Diabetes Mellitus Patients

**DOI:** 10.3390/nu9060573

**Published:** 2017-06-03

**Authors:** M. Gorety Jacobo-Cejudo, Roxana Valdés-Ramos, Ana L. Guadarrama-López, Rosa-Virgen Pardo-Morales, Beatriz E. Martínez-Carrillo, Laurence S. Harbige

**Affiliations:** 1 Faculty of Medicine, Universidad Autónoma del Estado de México, Paseo Tollocan esq. Jesús Carranza, Col. Moderna de la Cruz, Toluca 50180, Mexico; ln_gorejace@yahoo.com.mx (M.G.J.-C.); anag3075@hotmail.com (A.L.G.-L.); martinez_elina9@hotmail.com (B.E.M.-C.); 2 Instlituto Materno-Infantil del Estado de México, Paseo Colón s/n, Col. Villa Hogar, Toluca 50170, Mexico; rvpardo@gmail.com; 3 Faculty of Life Sciences and Computing, London Metropolitan University, 166-220 Holloway Road, London N7 8DB, UK; L.Harbige@londonmet.ac.uk

**Keywords:** type 2 diabetes mellitus, adipokines, lipid profile, *n*-3 PUFAs

## Abstract

Background: Type 2 diabetes mellitus (T2DM) is accompanied by chronic low-grade inflammation, with an imbalance in the secretion of adipokines and, worsening insulin resistance. Supplementation with *n*-3 PUFA in T2DM decreases inflammatory markers, the purpose of the study was to investigate the effect of *n*-3 PUFA supplementation on adipokines, metabolic control, and lipid profile in T2DM Mexican adults. Methods: In a randomized, single-blind, placebo-controlled pilot study, 54 patients with T2DM received 520 mg of DHA + EPA-enriched fish-oil (FOG) or a placebo (PG) daily. Baseline and 24-week anthropometric and biochemical measurements included glucose, insulin, glycosylated hemoglobin (Hb1Ac), leptin, adiponectin, resistin, and lipid profile; *n*-3 PUFA intake was calculated in g/day. Results: Waist circumference and blood glucose showed significant reductions in the FOG group (*p* = 0.001 and *p* = 0.011, respectively). Hb1Ac (*p* = 0.009 and *p* = 0.004), leptin (*p* < 0.000 and *p* < 0.000), and leptin/adiponectin ratio (*p* < 0.000 and *p* < 0.000) decreased significantly in both groups after 24 weeks (FOG and PG respectively). Serum resistin (FOG *p* < 0.000 and PG *p* = 0.001), insulin (FOG *p* < 0.000 and PG *p* < 0.000), and HOMA-IR (FOG *p* = 0.000 and PG *p* < 0.000) increased significantly in both groups. FOG had an overall improvement in the lipid profile with a significant decrease in triacylgycerols (*p* = 0.002) and atherogenic index (*p* = 0.031); in contrast, the PG group had increased total cholesterol (*p* < 0.000), non-HDL cholesterol (*p* < 0.000), and atherogenic index (*p* = 0.017). Conclusions: We found a beneficial effect of *n*-3 PUFA supplementation on waist circumference, glucose, Hb1Ac, leptin, leptin/adiponectin ratio, and lipid profile, without significant changes in adiponectin, and increases in resistin, insulin, and HOMA-IR in both groups.

## 1. Introduction

Type 2 diabetes mellitus (T2DM) is one of the most prevalent chronic diseases around the world, with an increase in its prevalence due to the increase in its risk factors such as obesity and physical inactivity [1]. According to the National Health and Nutrition Survey, in 2012, in Mexico there were 6.4 million people with T2DM, which means an increase in prevalence from 7% in 2006 to 9.2% in 2012 [2]. T2DM is characterized by impaired pancreatic β-cell function that causes impaired insulin secretion and insulin resistance (IR) mainly in liver, muscle, and adipose tissue [3].

It is well recognized that the actively secreted products of adipose tissue known as adipokines can modulate different functions [4]; adipokines play a pivotal role in the regulation of whole-body metabolism, as well as in inflammatory and immune responses, and are considered a link between obesity and the development of T2DM [5].

Adiponectin is a collagen-like protein exclusively expressed in adipose tissue [6], which possesses potent antidiabetic and anti-inflammatory properties [7]. In humans, plasma adiponectin levels are closely related to whole-body insulin sensitivity [8] and are correlated negatively with IR and T2DM. Prospective and longitudinal studies have indicated that lower adiponectin levels are associated with a higher incidence of T2DM [4]. Adiponectin regulates glucose and lipid metabolism through the reduction of fat storage (lipogenesis) and the promotion of fat utilization (fatty acid oxidation) [9].

Leptin is a cytokine-like molecule secreted by adipose tissue which regulates adipose tissue mass and body weight by inhibiting food intake and stimulating energy expenditure [6], thus maintaining energy homeostasis. Leptin correlates directly with adipose tissue mass. Obesity and T2DM are associated with increased plasma leptin levels, which fail to correct hyperglycemia in these patients because of the presence of leptin resistance [5], and these elevated plasma leptin levels are associated with IR, independent of obesity and insulin sensitivity [10].

In humans, resistin is mainly secreted by macrophages and monocytes and by organs such as spleen and bone marrow [11]. Since its discovery, resistin has been related to obesity and IR in many animal experiments, but the application of these findings to human studies has been difficult to determine. However, studies in humans have shown that serum resistin levels are higher in obese patients with T2DM compared with non-diabetic obese and that mRNA levels of resistin are higher in female patients with T2DM compared to healthy women [12].

Dyslipidemias are very common in type 2 diabetes mellitus and are the cause of cardiovascular disease in uncontrolled patients. Hypertriacylglyceridemia as well as low HDL-cholesterol concentrations, together with high LDL- and non-HDL-cholesterol is a common lipid profile pattern observed in subjects with diabetes [13,14,15,16].

Eicosapentaenoic acid (EPA) and docosahexaenoic acid (DHA) are polyunsaturated fatty acids found at high levels in fish oils [17]. Accumulating evidence suggests that *n*-3 PUFAs from fish oil may counteract the adipokine dysregulation that occurs in obesity and its related diseases like T2DM [9], but it is not well established if the consumption of *n*-3 PUFAs affects circulating adiponectin, resistin, and leptin in humans; the results are inconclusive [18]. Furthermore, research in humans and animal models show a lipid normalizing effect of PUFA supplementation [19,20].

For these reasons, we undertook a pilot study with the aim of investigating the effect of a six-month supplementation trial with *n*-3 PUFAs on adiponectin, resistin, leptin, and the lipid profile in adults with T2DM from Toluca, Mexico.

## 2. Materials and Methods

### 2.1. Study Design

This study was a randomized, single-blind and placebo-controlled pilot study conducted in eight Urban Public Health Centers (UPHC) in Toluca, Mexico, from February to September 2015. The inclusion criteria were the following: men and women with T2DM, between 25 and 60 years, without other chronic diseases such as hypertension, arthritis, kidney disease, cancer, and HIV, with a BMI ≤ 29.9, without fish allergies or insulin treatment, and not lactating or pregnant.

### 2.2. Methods

Sixty-five Mexican subjects with T2DM were enrolled from the eight UPHC in Toluca as follows: Capultitlán (*n* = 1), Emiliano Zapata (*n* = 6), Nueva Oxtototitlán (*n* = 2), Reforma (21), Seminario (3), Tlacotepec (1), San Diego de los Padres (*n* = 16), and Ejido de la Y (*n* = 15). The 65 subjects were randomly and blindly separated into an experimental group (fish oil group: FOG) of 34 patients (10 males, 24 females) and a control group (placebo group: PG) of 31 patients (5 males, 26 females). The majority of patients were medicated with Glibenclamide plus Metformin or Metformin alone and were controlled by their family practitioner, we did not give them additional dietary or lifestyle advice; we only asked them to continue with their regular medication and diet.

Written informed consent was given to all participants. The study was approved by the Research and Ethics Committee of the Universidad Autónoma del Estado de Mexico and the Institute for Health of the State of Mexico’s Research and Education Coordination. The protocol was conducted following the regulations of the Helsinki Declaration of 1975 and its amendment of 2013, as well as the principles for experiments with human beings from the Nüremberg Code for medical ethics. Individual data is being maintained confidential.

Once obtained the sample, the baseline evaluation was performed as follows:

#### Anthropometric evaluation:

All measurements were recorded with light clothes and height without shoes by a trained nutritionist who was previously standardized. Weight was measured in a TANITA^®^ scale (Mod. 1631) and was registered in kg; height was measured in cm using a portable SECA 206 stadiometer; the percentage of body fat was obtained by electrical bioimpedance with the same TANITA scale; body mass index (BMI) was calculated as body weight in kilograms divided by the square of height in meters. Waist and hip circumferences were measured with a Gülick™ fiberglass tape and the waist/hip ratio was calculated.

#### Biochemical evaluation:

After an overnight fast (≥12 h), collection of a venous blood sample into an 8.5 mL tiger-top tube containing gel and clotting activator for the subsequent biochemical analysis of glucose, insulin, adipokines, and lipid profile was undertaken. Another venous blood sample in a 3 mL tube with EDTA for the analysis of glycosylated hemoglobin was also obtained.

Serum was obtained after centrifugation at 900 *g* or 3400 rpm for 10 min at 4 °C, within 30 min after blood sample collection; serum samples for glucose and lipid profile were processed within one hour, and the rest of the samples were aliquoted and stored at −80 °C until assayed.

Serum glucose, triacylglycerols, total cholesterol, HDL-cholesterol, LDL-cholesterol, and glycosylated hemoglobin levels (in total blood) were measured by enzymatic colorimetric assays in the auto-analyzer Selectra II (ROCHE™), using reagents from RANDOX™ (Cat. GL1611, Cat. Tr213, Cat. CHO215, Cat. CH3811A, Cat. CH3811B and Cat. HA3830A). Non-HDL-cholesterol was calculated by difference (total cholesterol − HDL-cholesterol).

Serum levels of insulin were measured with a Human Insulin ELISA kit from EMD Millipore Corporation^®^ (Cat. # EZHI-14K) according to the manufacturer´s instructions. We evaluated IR with glucose and insulin concentrations using the Homeostatic model assessment as follows:

HOMA-IR = (fasting insulin (µU/mL)) × (fasting glucose (mg/dL))/405 [21].

Commercially available kits from Merck Millipore^®^ (Cat. # HADK1MAG-61K and HADK2MAG-61K) were used to measure the serum levels of adiponectin, resistin, and leptin in the Luminex^®^ 100™ analyzer by luminometry according to the manufacturer’s recommended protocols.

### 2.3. Supplementation

The supplementation period started after basal evaluation. Patients took two softgels per day of the assigned supplement for 24 weeks. Each 1.4 g *n*-3 PUFAs softgel contained a combination of 160 mg of eicosapentaenoic acid (EPA) with 100 mg of docosahexaenoic acid (DHA) from fish oil, so the total daily oral dose was 520 mg of *n*-3 PUFAs (320 mg of EPA and 200 mg of DHA, 2 g of total fat, 1.2 mg of vitamin E, gelatin and glycerine) during the six months of intervention. PUFA supplements were purchased from General Nutrition Centers™. The placebo softgels were identical to the *n*-3 PUFAs softgels in appearance and contained cornstarch (1 g carbohydrates and 1.7 mg of sodium per softgel).

### 2.4. Follow-up

After basal evaluation, patients were monitored by 6 monthly visits, in which they received their corresponding supplement and were followed-up every two weeks by telephone to make sure that they were taking their capsules, as well as to remind them not to change their habitual diet, medication, or physical activity regimes.

### 2.5. Diet Analysis

A trained nutritionist performed 24-h recalls, one at the beginning and one at the end of the study to analyze the diet. The dietary nutrient intake and the consumption of polyunsaturated fatty acids were calculated in grams and percentages using DIAL^®^ (ver 3.3.2., Universidad Complutense, Madrid, Spain) software. In the final 24 h recalls of the FOG, we added the nutritional composition of *n*-3 PUFA softgels.

### 2.6. Statistical Analysis

Data were expressed as mean ± SD. To compare differences in the same group after intervention, paired *t*-test was used for parametric variables and Wilcoxon signed rank test for non-parametric variables. In the diet analysis, unpaired two-sample *t*-test was used to compare differences between groups in a parametric way and a Mann–Whitney U-test for non-parametric data. Normality of data was assessed by the Kolmogorov–Smirnoff test. All analyses were performed using the statistical software SPSS (version 19.0; SPSS Inc., Chicago, IL, USA). A *p*-value < 0.05 was considered statistically significant.

## 3. Results

During the study, 10 subjects, 4 in the omega-3 group and 6 in the placebo group, were eliminated for not completing the six-month supplementation for personal reasons, and the drop-out rate in both groups was less than 20%. When analyzing the causes for drop-out, it is important to note that all patients had low-education and socioeconomic levels, and even though we tried to reach them by phone or house-visits, they were not located either to follow-up or to obtain data at the end of the trial. We analyzed basal data for differences between those who dropped out and those who stayed, and there were no significant differences between groups that indicated that they stopped attending due to the variables we were evaluating.

Age, gender distribution, and diabetes duration were similar between groups without significant differences; in the FOG, 7 subjects were males, and 22 were females; in the PG, 5 subjects were males and 20 were females. The mean age of the FOG was of 50.4 ± 6.3 years, while in the PG it was 48.1 ± 6.8 years (*p* = 0.208), and the average time of diabetes duration was of 6.6 ± 5.0 years in the FOG and of 6.5 ± 5.8 years in the PG (*p* = 0.954).

In anthropometric measurements, only the FOG showed a significant decrease in waist circumference after supplementation (*p* = 0.001) (Table 1). No significant changes in body weight (*p* = 0.250 and 0.578), BMI (*p* = 0.278 and 0.485), body fat (*p* = 0.889 and 0.614), and waist/hip ratio (*p* = 0.288 and 0.076) were observed in FOG and PG, respectively, after intervention.

At baseline, there was only a significant difference between groups in serum resistin levels (*p* = 0.006). The results of biochemical analysis are summarized in Table 2. Glucose serum levels only showed a significant decrease in FOG (*p* = 0.001), and there were significant reductions in glycosylated hemoglobin (*p* = 0.009 and *p* = 0.004), leptin (*p* = 0.000 and *p* = 0.000), and leptin/adiponectin ratio in both groups (FOG and PG, respectively). Adiponectin did not show significant changes (*p* = 0.177 for FOG and *p* = 0.563 for PG). Contrary to expected, resistin showed a significant increase in both groups (*p* = 0.000 for FOG and *p* = 0.001 for PG). With respect to IR, there were significant increases in insulin serum levels (*p* = 0.000 for FOG and *p* = 0.000 for PG) and in HOMA-IR (*p* = 0.000 for FOG and *p* = 0.000 for PG).

Table 2 shows changes in lipid profile indicators, FOG significantly decreased triacylglycerides (*p* < 0.01) and the atherogenic index (*p* < 0.05), whereas the placebo group showed a statistically significant increase in total cholesterol (*p* < 0.000), non-HDL-cholesterol (*p* < 0.000), and the atherogenic index (*p* < 0.05).

The results of diet analysis are shown in Table 3. The FOG showed a significant increase in total omega-3 fatty acids (*p* < 0.001), particularly in EPA and DHA (*p* < 0.000); *n*-6 to *n*-3 ratio decreased from 16:1 to 10:1 (*p* < 0.05). The placebo group showed a statistically significant increase in protein and monounsaturated fatty acid intake (*p* < 0.05). No differences were found in any other nutrient analyzed.

## 4. Discussion

In this study, we investigated the effect of supplementation with *n*-3 PUFAs on serum levels of adiponectin, resistin, and leptin in an adult Mexican population with T2DM.

The results of the anthropometric analysis (Table 1) are similar to the results observed in previous similar studies such as a study of supplementation with *n*-3 PUFAs in diabetic patients [22]; patients with impaired fasting glucose (IFG) or impaired glucose tolerance (IGT) [23]; and two more in women with polycystic ovarian syndrome [24,25], which regularly present insulin resistance. All of these studies showed no significant change in body weight, BMI, body fat, waist circumference, or waist/hip ratio after the supplementation period, even at higher doses of *n*-3 PUFAs and longer periods of supplementation. However, in a crossover model in which 16 T2DM patients were assigned to one of two consecutive 3.5-week periods of diabetic diets (foods rich in *n*-6 or *n*-3 PUFAs), the authors found a slight but significant reduction in body weight and BMI in both dietary periods [26]. This suggests that *n*-3 PUFAs from food are more effective in controlling body weight than *n*-3 PUFAs from supplements. In our study, supplementation with *n*-3 PUFAs for 24 weeks only helped to significantly reduce waist circumference and did not help control body weight or reduce BMI, body fat percentage, or waist/hip ratio. Furthermore, one of the studies in women with polycystic ovarian syndrome reported that effects of *n*-3 PUFAs on anthropometric measurements might be dependent on gender, age, and BMI of subjects at baseline [25].

The effect of *n*-3 PUFAs on glucose and glycosylated hemoglobin is not clear. A study of supplementation with DHA-rich fish oil in an Iranian diabetic population [27] and reviews of clinical trials of supplementation with *n*-3 PUFAs in different diabetic populations, have not found significant positive effects on glycemic control [17,28]. However, a recent study [23] showed that *n*-3 PUFA supplementation with high doses for 18 months was effective in reducing glycemia and IR in patients affected by IFG or IGT and that the regression of the condition of impaired glycemia to normoglycemia seems to be helpful in delaying the development of T2DM. Flanchs et al. showed in a review that the beneficial effects of *n*-3 PUFAs on insulin sensitivity and glucose metabolism in people with IR are dependent on factors such as disease progression and age [29]. In this study, we found a significant positive effect of *n*-3 PUFA supplementation on glycemic control (glucose and glycosylated hemoglobin), but more studies are needed to test this hypothesis because most of the beneficial effects have been shown in epidemiologic studies based on a habitual fish diet consumed for years [28].

As expected, there were significant reductions in leptin serum levels and hence in leptin/adiponectin ratio, which has been demonstrated to be related to obesity and T2DM [29]. Contrary to expectation, adiponectin did not increase significantly, while resistin increased significantly. Our results are not in line with the results observed in similar studies of supplementation with *n*-3 PUFAs in diabetes [8] and in healthy subjects with a high consumption of fish, which has shown significant increases in adiponectin serum levels after various supplementation periods [30]. As the changes in adipokine profile were similar in both groups, we cannot affirm that higher doses of *n*-3 PUFAs would have led to a decrease in leptin and leptin/adiponectin ratio because there was no lead tendency toward lower leptin concentrations in subjects with the *n*-3 PUFA supplement than in subjects with the placebo.

Our study population showed higher basal adiponectin and leptin serum levels than have been reported in other similar studies, for example, in German [31], Finnish [32], Japanese [8], Asian Indian [33], and Arabic diabetic populations [34], which is likely to be due to ethnicity. Resistin showed similar results compared with serum resistin levels of Egyptians and Japanese although slightly lower [35,36].

It has been demonstrated that weight reduction increases plasma adiponectin concentrations and improves IR [10], but in this study, possibly because there were no significant changes in body weight, there were no significant increases in serum adiponectin levels. In a study with *ob*/*ob* mice, the administration of recombinant adiponectin, even after the development of diabetes, significantly ameliorated hyperglycemia [37], and it has been reported that serum adiponectin levels correlate inversely with IR [5]. Plasma adiponectin levels have also been demonstrated to be negatively correlated to IR [4], but in this study, we did not find a negative correlation because adiponectin (non-significant) and HOMA-IR increased.

Leptin correlates directly with adipose tissue mass; [5] however, we did not observe a significant reduction in body weight, BMI, and body fat, despite the reduction in serum leptin levels. When we used a bivariate correlation analysis, we found that serum leptin levels showed a positive correlation with BMI (*R* = 0.353 and *p* = 0.009) and body fat percentage (*R* = 0.518 and *p* = 0.000), which is in line with the results of a similar study [5].

Leptin/adiponectin ratio showed a significant reduction in both groups related to the significant decrease in leptin levels. Leptin/adiponectin ratio was a useful measure of IR in non-diabetic white adults [38], but we did not observe a positive correlation between HOMA-IR and leptin serum levels; despite the reduction in this ratio, there was no reduction in HOMA-IR. However, these findings suggest the use of the leptin/adiponectin ratio as a useful tool to detect IR. On the other hand, resistin has been linked to obesity and IR since its discovery [12], but our results are not consistent with these findings because we found a weak negative correlation between resistin levels and HOMA-IR (*R* = −0.274 and *p* = 0.045).

Obesity is the most significant factor contributing to IR and T2DM [39], and although our patients were not obese, we found a weak but significant positive correlation between body fat percentage and HOMA-IR (*R* = 0.275 and *p* = 0.046). This factor might explain the significant increase in HOMA-IR in both groups because there was a non-significant increase in body fat percentage.

Our results are not in accordance with those of Yamamoto et al., who showed in a supplementation study with EPA in hyperlipidemic patients that IR determined by the HOMA-IR was significantly improved in the supplemented group, compared with the placebo [21]. In our study, both groups showed an increase in HOMA-IR, more studies are therefore necessary to evaluate only the EPA effect in the IR of individuals with T2DM.

Our data show an overall improvement in the lipid profile through the atherogenic index in the *n*-3 PUFA supplemented group, with an overall deterioration in the placebo group. Derosa et al. found an increase in HDL-cholesterol and a decrease in triacylglycerides after 18 months of supplementation with *n*-3 PUFA, in patients with impaired glucose metabolism [23]. While in subjects with type 2 diabetes supplemented with DHA-rich fish oil, Mansoori et al. found a decrease in triacylglycerides independently of their initial values [27]. However, several trials of supplementation with *n*-3 PUFA have not found an effect on lipid profile in adults with T2DM [40,41].

Non-HDL-cholesterol has been recommended as a sensitive proxy of the atherogenic metabolic lipid status; importantly, its values in our study population showed a non-significant decrease in the supplemented group but a highly significant increase in the placebo group. This may be considered a protective effect of *n*-3 PUFA supplementation on the lipid metabolic deterioration caused by T2DM [15,16].

With respect to diet analysis, our results are in line with those of a previous study of this group that showed that a Mexican population with T2DM had a very low intake of *n*-3 PUFAs and a high consumption of lipids, particularly saturated fatty acids [42]. According to the recommendations of the Mexican Official Standard NOM-015-SSA2-2010, for prevention, treatment, and control of diabetes mellitus [43], the mean daily energy intake of all groups was adequate, though protein and *n*-3 PUFA intake in both groups and carbohydrate intake in FOG were lower than recommendations, while the total lipid intake and saturated fatty acid intake were higher than recommendations in FOG.

It is well known that the quality of dietary fat is a key determinant of IR and that saturated and trans fatty acids decrease insulin secretion and worsen insulin sensitivity [10]; a factor which could explain the higher increase in HOMA-IR in FOG because of their higher intake of saturated fatty acids.

The quality of fat may have a significant influence on adiponectin concentrations. The data from 13 studies in a systematic review showed a modest and significant effect of *n*-3 PUFA supplementation on adiponectin concentrations [44]. Because of the very low intake of *n*-3 PUFAs in both our groups, we may not have been able to observe significant changes in adiponectin levels. *In vitro* human adipocyte studies found that EPA and DHA (100 μM) treatment for 48 h, increases adiponectin secretion, and that only EPA led to higher cellular adiponectin being introduced into the adipocytes, suggesting that the regulation of adiponectin by *n*-3 PUFAs is dose- and time-dependent and that it can be affected by the maturation stage of adipocytes [9]. However, another study of supplementation with *n*-3 PUFAs in Mexican children and adolescents with obesity and insulin resistance, showed that for *n*-3 PUFAs, the duration of treatment is not associated with the effects observed and that the dosage could be more important, which in this case would suggest that in our study the *n*-3 PUFA dose we used can be considered low [45].

It has been demonstrated that diets high in fish and flaxseed oil lower the serum phospholipid ratio of omega-6/omega-3 fatty acids and that *n*-3 PUFAs are beneficial in patients affected by inflammatory diseases. The omega 6/omega 3 ratio is of importance in health and disease; during the Paleolithic period of human evolution, there was a different balance between *n*-6 and *n*-3, but the last 150 years have seen a significant increase, as in Western diets this ratio is now in the range of 15–20:1 [46]. Because of the low intake of *n*-3 PUFAs and the increased amounts of the *n*-6 PUFA linoleic acid (and high levels found in plasma phospholipids to be published elsewhere) in our study population, the omega 6/omega 3 ratios were of 11:1 and of 13:1 in FOG and PG, respectively, very similar to Western diets [47].

Although we found a beneficial effect of EPA + DHA supplementation on waist circumference, glucose, glycosylated hemoglobin, leptin, and leptin/adiponectin ratio in this population, these beneficial effects may not have been due to the supplement alone because we observed similar results in some of these parameters between patients who took the *n*-3 PUFA supplement and those that took the placebo. Similarly, there were significant increases in resistin, serum insulin, and HOMA-IR in both groups. It is possible that some of the beneficial effects observed in both groups were due to metformin and that the combined use of *n*-3 PUFA and metformin gave rise to better outcome measures. However, the significant increase in monounsaturated fatty acids and protein in the placebo group may also have contributed to the decrease in glycosylated hemoglobin, leptin, and leptin/adiponectin ratio and the increase in resistin, insulin, and HOMA-IR in this group. Interestingly, Schwingshack et al., 2001, and McAllan et al., 2014, have reported that macronutrient quality and composition, i.e. monounsaturated fat and protein can affect some of the parameters we have measured (leptin and glycosylated hemoglobin) and in the same direction we observed for the placebo group [48,49].

## 5. Conclusions

Additional research is required to fully understand the associated mechanisms between *n*-3 PUFA-rich fish oil consumption and adipokine secretion/expression. The small sample size and use of metformin may have limited our findings as well as the use of a relatively low dose of *n*-3 PUFAs, so larger studies with higher doses of *n*-3 PUFAs are required to confirm and extend our findings. It would be important to measure serum levels of other adipokines related to IR and T2DM, such as visfatin and apelin, to determine if the beneficial effects of PUFA *n*-3 are sustained after the cessation of therapy and to determine if negative effects on insulin sensitivity are actually caused by the supplement. The different effects of EPA and DHA observed in some studies highlight the need to study their actions separately.

In summary and despite the limitations, our study is the first to report serum concentrations of adiponectin, resistin, and leptin and show a beneficial effect of *n*-3 PUFA supplementation on waist circumference, glucose, glycosylated hemoglobin, leptin, and leptin/adiponectin ratio and lipid profile in a group of Mexican individuals with T2DM. Supplementation with *n*-3 PUFA and *n*-3 PUFA combined with metformin both warrant further investigation in Mexican T2DM.

## Figures and Tables

**Table 1:** Anthropometric measurements.

	Fish Oil Group (*n* = 29)			Placebo Group (*n* = 25)		
Variables	Basal	Final	*p*	Basal	Final	*p*
Body weight (kg)	63.0 ± 9.3	62.7 ± 9.4	0.250	60.2 ± 6.4	60.0 ± 7.3	0.578
BMI (kg/m^2^)	25.6 ± 2.4	25.4 ± 2.7	0.278	26.0 ± 1.6	25.9 ± 2.0	0.485
Body fat (%)	30.9 ± 9.1	31.1 ± 7.2	0.889	29.9 ± 5.3	30.4 ± 6.1	0.614
Waist circumference (cm)	86.4 ± 7.6	83.1 ± 6.2	0.001	83.2 ± 5.3	83.1 ± 6.1	0.893
Waist/hip ratio	0.89 ± 0.05	0.90 ± 0.05	0.288	0.90 ± 0.05	0.92 ± 0.04	0.076

BMI: body mass index. Paired *t*-test was performed to compare differences in time (before and after supplementation). A *p*-value < 0.05 was considered statistically significant, marked in bold numbers.

**Table 2:** Biochemical measurements.

	Omega-3 (*n* = 29)			Placebo (*n* = 25)		
Variables	Basal	Final	*p*	Basal	Final	*p*
Glucose (mg/dL) ^‡^	177.2 ± 68.4	156.1 ± 69.4	0.011	184.6 ± 71.1	183.3 ± 53.3	0.326
Glycosylated hemoglobin (%) ^†^	9.6 ± 3.1	8.2 ± 1.9	0.009	10.0 ± 2.1	9.0 ± 1.8	0.004
Adiponectin (µg) ^‡^	23.6 ± 20.3	24.5 ± 13.0	0.177	22.8 ± 10.5	24.3 ± 13.3	0.563
Leptin (ng) ^†^	21.7 ± 15.5	3.9 ± 2.5	0.000	18.4 ± 13.2	3.5 ± 2.3	0.000
Resistin (ng) ^‡^	30.2 ± 14.0	65.9 ± 23.4	0.000	39.2 ± 12.5	61.3 ± 20.6	0.000
Leptin/adiponectin ratio ^†^	1.3 ± 1.2	0.24 ± 0.26	0.000	0.88 ± 0.68	0.17 ± 0.12	0.000
Insulin µU/mL ^‡^	7.6 ± 3.0	14.2 ± 8.2	0.000	6.5 ± 1.6	10.2 ± 3.3	0.000
HOMA-IR ^‡^	3.1 ± 1.3	5.3 ± 3.8	0.000	2.9 ± 1.2	4.4 ± 1.6	0.000
Total Cholesterol (mg/dL) ^†^	203.38 ± 33.72	199.10 ± 47.63	0.542	180.32 ± 30.56	209.75 ± 36.80	0.000
Triacylglycerides (mg/dL) ^†^	186.24 ± 85.58	137.28 ± 65.39	0.002	269.40 ± 169.30	251.20 ± 149.76	0.503
HDL-Cholesterol (mg/dL) ^†^	43.52 ± 7.95	48.13 ± 14.59	0.076	38.35 ± 9.51	40.01 ± 9.28	0.384
LDL-Cholesterol (mg/dL) ^†^	131.00 ± 34.66	129.82 ± 44.74	0.869	109.40 ± 34.22	127.26 ± 38.80	0.076
Non-HDL-Cholesterol (mg/dL) ^†^	159.52 ± 31.28	150.97 ± 44.26	0.152	141.97 ± 24.98	169.74 ± 38.15	0.000
Atherogenic Index ^†^	5.04 ± 1.77	4.37 ± 1.04	0.031	4.86 ± 1.06	5.52 ± 1.63	0.017

^†^ Paired *t*-test was used for parametric variables and ^‡^ Wilcoxon signed rank test for non-parametric variables. A *p*-value ≤ 0.05 was considered statistically significant, marked in bold numbers.

**Table 3:** Diet analysis.

Variable	Omega-3 (*n* = 29)			Placebo (*n* = 25)		
	Basal	Final	*p*	Basal	Final	*p*
Energy (kcal/day)	1562.2 ± 387.4	1672.8 ± 665.2	0.476	1751.4 ± 479.9	1875.9 ± 698.5	0.276
Protein (g/day)	57.3 ± 17.2	59.4 ± 24.3	0.737	50.8 ± 19.4	60.5 ± 27.8	* 0.030
Carbohydrates (g/day)	192.1 ± 60.4	187.8 ± 68.8	0.729	258.2 ± 73.1	255.0 ± 104.7	0.905
Lipids (g/day)	59.5 ± 21.2	72.2 ± 38.4	0.097	54.2 ± 27.7	65.2 ± 31.1	0.092
Saturated fatty acids (g/day)	16.4 ± 7.2	19.3 ± 11.9	0.271	11.4 ± 9.0	14.7 ± 9.7	0.065
Monounsaturated fatty acids (g/day)	20.2 ± 8.2	24.6 ± 15.5	0.160	15.4 ± 10.6	22.6 ± 14.3	* 0.013
Polyunsaturated fatty acids (g/day)	13.2 ± 8.1	16.5 ± 12.7	0.197	13.2 ± 11.9	13.0 ± 6.4	0.367
Omega-3 fatty acids (g/day)	0.89 ± 0.76	1.32 ± 0.34	* 0.001	0.79 ± 1.12	0.75 ± 0.53	0.178
Omega-6 fatty acids (g/day)	10.5 ± 7.7	13.6 ± 12.0	0.221	9.4 ± 11.0	8.8 ± 5.2	0.382
*n*6:*n*3 Ratio	14:1	10:1	* 0.025	16:1	14:1	0.882
EPA (g/day)	0.077 ± 0.317	0.330 ± 0.018	* 0.000	0.016 ± 0.037	0.047 ± 0.177	0.837
DHA (g/day)	0.103 ± 0.236	0.287 ± 0.089	* 0.000	0.086 ± 0.109	0.155 ± 0.187	0.074

Mann–Whitney U test for differences between groups. * A *p*-value < 0.05 was considered significant, marked with bold numbers.
